# Supplementary material for: Genome of the endangered eastern quoll (Dasyurus viverrinus) reveals signatures of historical decline and pelage color evolution
Source: Commun Biol. 2024 May 25;7:636. doi: 10.1038/s42003-024-06251-0 (PMC11128018; doi:10.1038/s42003-024-06251-0)
Supplement: Supplementary file 2 — Supplementary Information [file 42003_2024_6251_MOESM2_ESM.pdf]

Supplementary Table 1: Eastern quoll chromosome homology with related dasyurids based on gene annotation overlap					
**Eastern Quoll DasViv_1.0 vs Tasmanian Devil mSarHar1.11**					
**Chromosome Number**	**Quoll Scaffold ID**	**Devil Scaffold ID**	**Quoll Scaffold Length (nt)**	**Devil Scaffold Length (nt)**	**Gene Annotation Overlap Percentage**
1	CM036997.1	NC_045426.1	714541192	716413629	99.62
2	CM036998.1	NC_045427.1	662896076	662751787	99.68
3	CM036999.1	NC_045428.1	628486596	611347268	99.52
4	CM037000.1	NC_045429.1	468983349	464895054	99.24
5	CM037001.1	NC_045430.1	292278231	288121652	99.39
6	CM037002.1	NC_045431.1	255917514	254895979	99.33
X	CM037003.1	NC_045432.1	95405421	83081154	98.09
**Eastern Quoll DasViv_1.0 vs Yellow-Footed Antechinus AdamAnt_v2**					
**Chromosome Number**	**Quoll Scaffold ID**	**Antechinus Scaffold ID**	**Quoll Scaffold Length (nt)**	**Antechinus Scaffold Length (nt)**	**Gene Annotation Overlap Percentage**
1	CM036997.1	NC_067398.1	714541192	727308865	97.47
2	CM036998.1	NC_067399.1	662896076	686337178	98.26
3	CM036999.1	NC_067400.1	628486596	636717922	98.28
4	CM037000.1	NC_067401.1	468983349	486195733	98.48
5	CM037001.1	NC_067402.1	292278231	300479852	98.50
6	CM037002.1	NC_067403.1	255917514	264590327	98.13
X	CM037003.1	NC_067404.1	95405421	86570782	95.61

Supplementary Table 2: BUSCO gene recovery from marsupial genome assemblies					
**BUSCO v5.4.2, mammalia_odb10 (9226 reference orthologs)**					
**Species**	**Assembly Version**	**Complete Single-Copy**	**Complete and Duplicated**	**Fragmented**	**Missing**
antechinus flavipes	GCF_016432865.1_AdamAnt_v2	8386	139	106	595
antechinus stuartii	GCA_016696395.1_USYD_AStu_M	8329	187	153	557
bettongia penicillata	GCA_023548195.1_mBetpen1.pri.20210916	8452	236	93	445
dasyurus viverrinus	GCA_020854095.1_UniMelb_DasViv_v1.0	8503	124	109	490
dromiciops gliroides	GCF_019393635.1_mDroGli1.pri	8316	341	95	474
gracilinanus agilis	GCF_016433145.1_AgileGrace	7884	243	280	819
gymnobelideus leadbeateri	GCA_011680675.1_LBP_v1	7534	772	270	650
lagorchestes hirsutus	GCA_028533205.1_Lagorchestes_hirsutus_HiC	8040	243	251	692
macropus eugenii	GCA_028372415.1_mMacEug1.pri	8468	266	82	410
macropus fuliginosus	GCA_028583105.1_mf-2k	7585	179	447	1015
macropus giganteus	GCA_028627215.1_mg-2k	7988	183	286	769
monodelphis domestica	GCF_000002295.2_MonDom5	7925	220	181	900
myrmecobius fasciatus	GCA_023553655.1_mMyrfas1.20211206	6758	502	577	1389
petaurus breviceps	GCA_028583685.1_PetGlider_PUasm1.0	7986	592	131	517
phalanger gymnotis	GCA_028646595.1_pg-2k	7349	318	471	1088
phascolarctos cinereus	GCF_002099425.1_phaCin_unsw_v4.1	8529	151	115	431
potorous gilbertii	GCA_028658325.1_Potorous_gilbertii_HiC	6906	953	380	987
pseudocheirus occidentalis	GCA_028646575.1_Pseudocheirus_occidentalis_HiC	6663	458	631	1474
pseudochirops corinnae	GCA_028646515.1_Pseudochirops_corinnae_HiC	6748	438	608	1432
pseudochirops cupreus	GCA_028627135.1_Pseudochirops_cupreus_HiC	7345	274	494	1113
sarcophilus harrisii	GCF_902635505.1_mSarHar1.11	8336	114	140	636
thylacinus cynocephalus	GCA_007646695.3_UniMelb_ThyCyn2.0_hybrid_assembly	7602	77	372	1175
trichosurus vulpecula	GCF_011100635.1_mTriVul1.pri	8282	390	94	460
vombatus ursinus	GCF_900497805.2_bare-nosed_wombat_genome_assembly	8298	307	119	502

Supplementary Table 3: Eastern quoll RNA-seq libraries generated for gene annotation (BioProject: PRJNA963007)			
**Tissue**	**SRA Accession**	**Read Pairs Pre-Filtering**	**Read Pairs Post-Filtering**
Primary Fibroblasts	SRR24349374	58106149	40666044
Heart	SRR24349373	56520965	39690734
Kidney	SRR24349372	58208847	29277865
Liver	SRR24349371	66155670	34400334
Spleen	SRR24349370	51661979	35943540

Supplementary Table 4: Homologous protein sets used for annotating the eastern quoll genome		
**Species**	**Assembly**	**Number of Proteins**
Monodelphis domestica	GCF_000002295.2_MonDom5	49112
Phascolarctos cinereus	GCF_002099425.1_phaCin_unsw_v4.1	46908
Trichosurus vulpecula	GCF_011100635.1_mTriVul1.pri	35542
Antechinus flavipes	GCF_016432865.1_AdamAnt_v2	40823
Gracilinanus agilis	GCF_016433145.1_AgileGrace	28926
Vombatus ursinus	GCF_900497805.2_bare-nosed_wombat_genome_assembly	42624
Sarcophilus harrisii	GCF_902635505.1_mSarHar1.11	45893

Supplementary Table 5: Detailed summary of repeat annotations from RepeatMasker				
**Interspersed Repeats**				
**Major Repeat Class**	**Specific repeat class**	**Element count**	**Number of nucleotides**	**Percent of assembly**
DNA Transposons	hAT-Charlie	223930	30567922	0.98%
	TcMar-Tigger	113443	18010462	0.58%
	hAT-Tip100	57751	9327442	0.30%
	TcMar-Tc1	9748	2004929	0.06%
	DNA	11969	1579017	0.05%
	TcMar-Mariner	3218	647061	0.02%
	hAT-Tag1	2184	545981	0.02%
	hAT	2910	506468	0.02%
	hAT-Ac	2624	300392	0.01%
	PIF-Harbinger	575	90718	0.00%
	Kolobok	520	83960	0.00%
	hAT-Blackjack	647	57927	0.00%
	Crypton	341	49422	0.00%
	TcMar	220	38565	0.00%
	Merlin	252	30285	0.00%
	Crypton-A	220	30232	0.00%
	TcMar-Pogo	45	5546	0.00%
	TcMar-Tc2	47	5471	0.00%
	hAT-hAT19	25	2251	0.00%
	PiggyBac	14	1365	0.00%
	CMC-Chapaev-3	4	403	0.00%
LINE	L1	874272	629441545	20.15%
	L2	656540	171423114	5.49%
	CR1	324025	91150392	2.92%
	RTE-RTE	101107	40127238	1.28%
	RTE-BovB	56198	23470315	0.75%
	RTE-X	1434	456712	0.01%
	Dong-R4	705	163354	0.01%
	L1-Tx1	208	74456	0.00%
	Penelope	750	68649	0.00%
	I-Jockey	99	15412	0.00%
	Jockey	2	81	0.00%
LTR	ERVK	63546	41337030	1.32%
	ERV1	81124	16179425	0.52%
	LTR	16720	4943618	0.16%
	Gypsy	5777	2328462	0.07%
	ERVL	1771	378950	0.01%
	KERV	86	7906	0.00%
	ERVL-MaLR	83	4880	0.00%
	ERV-Lenti	17	893	0.00%
	ERV-Foamy	16	815	0.00%
Other	Other	8	858	0.00%
RC	Helitron	677	108157	0.00%
Retroposon	L2-derived	105695	14426867	0.46%
	SVA	14	1232	0.00%
SINE	MIR	1851862	239545924	7.67%
	RTE-BovB	75434	19449979	0.62%
	tRNA-Core-RTE	132298	18559033	0.59%
	tRNA-RTE	6314	866127	0.03%
	5S-Deu-L2	2606	282085	0.01%
	tRNA	2027	266964	0.01%
	tRNA-Deu	505	56004	0.00%
	7SL	93	11592	0.00%
	Alu	45	2744	0.00%
	tRNA-C	18	931	0.00%
	5S	8	682	0.00%
	B4	9	444	0.00%
	tRNA-7SL	8	399	0.00%
	ID	8	374	0.00%
	Core-RTE	2	228	0.00%

	tRNA-Deu-L2	1	77	0.00%
	B2	2	74	0.00%
	RTE	1	38	0.00%
ARTEFACT	ARTEFACT	4	187	0.00%
Segmental	Segmental	2	110	0.00%
Unknown	Unknown	5051	781273	0.03%
**Other Repeats**				
Low_complexity	Low_complexity	194260	17051380	0.55%
RNA	RNA	99	17241	0.00%
Satellite	Satellite	5929	1948973	0.06%
	Y-chromosome	6	363	0.00%
	centr	5	334	0.00%
	telo	2	106	0.00%
Simple_repeat	Simple_repeat	1220645	74387023	2.38%
scRNA	scRNA	13723	2885085	0.09%
rRNA	rRNA	1010	139245	0.00%
tRNA	tRNA	1683	114825	0.00%
snRNA	snRNA	990	91798	0.00%
srpRNA	srpRNA	26	3360	0.00%

**Total:**	**6236237 elements**	**1.476 gigabases**	**47.2% of assembly length**

Supplementary Table 6: Reference genomes of other dasyuromorphs used in comparative genomics			
**Species**	**Common Name**	**Assembly**	**Accessed From**
Myrmecobius fasciatus	Numbat	Myrmecobius_fasciatus_HiC	DNA Zoo
Thylacinus cynocephalus	Thylacine	GCA_007646695.3_UniMelb_ThyCyn2.0_hybrid_assembly	GenBank
Antechinus stuartii	Brown antechinus	GCA_016696395.1_USYD_AStu_M	GenBank
Antechinus flavipes	Yellow-footed antechinus	GCF_016432865.1_AdamAnt_v2	GenBank
Dasyurus viverrinus	Eastern quoll	GCA_020854095.1_UniMelb_DasViv_v1.0	Present Study
Sarcophilus harrisii	Tasmanian devil	GCF_902635505.1_mSarHar1.11	GenBank
