# Supplementary material for: Genome of the endangered eastern quoll (Dasyurus viverrinus) reveals signatures of historical decline and pelage color evolution
Source: Commun Biol. 2024 May 25;7:636. doi: 10.1038/s42003-024-06251-0 (PMC11128018; doi:10.1038/s42003-024-06251-0)
Supplement: Supplementary file 3 — Description of Additional Supplementary Files [file 42003_2024_6251_MOESM3_ESM.pdf]

Description of Additional Supplementary Files

File name: Supplementary Data 1
Description: Alignment of dasyuromorph ASIP orthologs

File name: Supplementary Data 2
Description: Alignment of ASIP exon 1 region
