# Supplementary material for: Genome of the endangered eastern quoll (Dasyurus viverrinus) reveals signatures of historical decline and pelage color evolution
Source: Commun Biol. 2024 May 25;7:636. doi: 10.1038/s42003-024-06251-0 (PMC11128018; doi:10.1038/s42003-024-06251-0)
Supplement: Supplementary file 4 — Supplementary Data 1 [file 42003_2024_6251_MOESM4_ESM.pdf]

Supplementary Data 1: Alignment of dasyuromorph ASIP orthologs

Mfas – Numbat (*Myrmecobius fasciatus*)

Tcyn – Thylacine (*Thylacinus cynocephalus*)

Astu – Brown antechinus (*Antechinus stuartii*)

Afla – Yellow-footed antechinus (*Antechinus flavipes*)

Dviv – Eastern quoll (*Dasyurus viverrinus*)

Shar – Tasmanian devil (*Sarcophilus harrisii*)

Missing sequence in the Tasmanian devil

Alignment Of ASIP protein - MAFFT (v7.511)

```
Mfas        MTAKHLFLPFLLACLWFLAAYCHLAEEEKWSKDKGLGRNSMNLTDFPSVSIVALNKKSKK
Tcyn        MTAKHLLLPFLLVCLWFLAAYCHLAEEEKWSKDRSLGRNSMNLPDFPSVSIVALNKKSKK
Astu        MTAKHLFLPFLLACLWFLAAYCHLAEEEKWSKDRGLGRSSMNLPDFPSVSIVALNKKSKK
Afla        MTAKHLFLPFLLACLWFLAAYCHLAEEEKWSKDRSLGRSSMNLPDFPSVSIVALNKKSKK
Dviv        MTAKHLFLPFLLACLWFLAAYCHLAEEEKWSKDRGLGRSSMNLPDFPSVSIVALNKKSKK
Shar        -----------------------------------------------------LNKKSKK
                                                                 *******

Mfas        SIRKEIETKKSSEKKAVVKKSPSRSNCAATGAFCQPHTLSCCEPCASCYCRFFGRVCSCR
Tcyn        SIRKEIETKKSSEKKAVVKKSSSGSNCAATGAFCQPQTLSCCNRCATCHCRFFRSSCSCR
Astu        SIRKEIETKKSSEKKAVVKKSSSASTCAATGAFCQPQTISCCDKCDTCHCRFFGSVCFCR
Afla        SIRKEIETKKSSEKKAVVKKSSSASTCAATGAFCQPQTISCCDKCDTCHCRFFGSVCFCR
Dviv        NIRKEIETKKSSEKKAVVKKSSSASTCAATGAFCQPQTISCCNKCDTCHCRFFGSVCFCR
Shar        NIRKEIETKKSSEKKPVVK--LSASTCAATGAFCQPQTISCCNKCDTCHCRFFGSVCFCR
            .**************.***   * *.**********:*:***: * :*:****   * **

Mfas        LFQQSC
Tcyn        LFQPGC
Astu        PFMRKC
Afla        PFMRKC
Dviv        QFLRKC
Shar        PFLRKC
             *   *
```
