# Supplementary material for: Genome of the endangered eastern quoll (Dasyurus viverrinus) reveals signatures of historical decline and pelage color evolution
Source: Commun Biol. 2024 May 25;7:636. doi: 10.1038/s42003-024-06251-0 (PMC11128018; doi:10.1038/s42003-024-06251-0)
Supplement: Supplementary file 5 — Supplementary Data 2 [file 42003_2024_6251_MOESM5_ESM.pdf]

Supplementary Data 2: Alignment of *ASIP* exon 1 region

Shar 1 - mSarHar1.11 (Region = NC_045427.1:421297207-421297632)

Shar 2 - SarHar_Dovetail_2.0 (Region = VOSF01009762.1:640079-640504)

Afla - AdamAnt_v2 (Region = NC_067399.1:439018275-439020331)

Dviv - UniMelb_DasViv_v1.0 (Region = CM036998.1:421733752-421736969)

ASIP exon 1

Putative eastern quoll insertion boundaries (10bp highlighted)

Putative Tasmanian devil deletion boundaries (10 bp highlighted)

```
Alignment Of ASIP Exon 1 Region - MAFFT (v7.511)

Shar1       tcttacccatcttttaatgggtattaaataccgaattctctag-aagccttctttgaaac
Shar2       tcttacccatcttttaatgggtattaaataccgaattctctag-aagccttctttgaaac
Dviv        tcttacccatcttttaatgggtattaaataccgaattctctagaaagccttctttgaaac
Afla        tcttacccatcttttaatgaatattaaatactgaatcctctagaaagccttctttgaaac
            *******************..**********.****.****** ****************

Shar1       agtctccctttacactgtccatatcttgtattttcataattatctgcaagttgcctcctc
Shar2       agtctccctttacactgtccatatcttgtattttcataattatctgcaagttgcctcctc
Dviv        attctccctttacactgtccctatcttgtattttcataattatctacaagttgcctcctc
Afla        agtctccctttacactgttcatatcttgtattttcataattatctgcaagttgcctcctc
            * ****************.* ************************.**************

Shar1       cattagaaggtgaattccttgagagtaggaaattaggctttttcctttccttaaaacccc
Shar2       cattagaaggtgaattccttgagagtaggaaattaggctttttcctttccttaaaacccc
Dviv        cattagaaggtgaattccttgagagtaggaaa-taggctttttcctttccttaaaacccc
Afla        tattagaacatgaattccttgagagtaggaaattaggctttttcgtttccttaaattccc
            .******* .********************** *********** ********** .***

Shar1       acggttaagggcagtggctg----------------------------------------
Shar2       acggttaagggcagtggctg----------------------------------------
Dviv        agggttaagggcagtgtgtgtgtgtatatatatatatatatatatatatacatacaca
Afla        agggataagggcagtggctg----------------------------------------
            * ** ***********  **

Shar1       -----gcacatagtatcaatcaatcaat--------------------------------
Shar2       -----gcacatagtatcaatcaatcaat--------------------------------
Dviv        cacacacacatagtatcaatcaatcaataaacatttattgtgtctacacacctgtcagat
Afla        -----gcacatagtatcaatcaatcaat--------------------------------
                 .**********************
```

```
Shar1       ------------------------------------------------------------
Shar2       ------------------------------------------------------------
Dviv        tggctaagatgacaggaaaaaataataatgattgttggaggggatgtgggaaaactggga
Afla        ------------------------------------------------------------


Shar1       ------------------------------------------------------------
Shar2       ------------------------------------------------------------
Dviv        cattgttgcattgttggtggagttgtgaacgaatccaaccattttggagagttgtttgga
Afla        ------------------------------------------------------------


Shar1       ------------------------------------------------------------
Shar2       ------------------------------------------------------------
Dviv        actatgctcaaaaagttatcaaactgtgcatacccttgatccagcagtgttactactgg
Afla        ------------------------------------------------------------


Shar1       ------------------------------------------------------------
Shar2       ------------------------------------------------------------
Dviv        gcttatatcccaaagagattataaagcagggaaagggacctgtatgtgcacgaatgtttg
Afla        ------------------------------------------------------------


Shar1       ------------------------------------------------------------
Shar2       ------------------------------------------------------------
Dviv        tggcagccctttttgtagtggctagaaactggaagctgaatggatgcccatcagttggag
Afla        ------------------------------------------------------------


Shar1       ------------------------------------------------------------
Shar2       ------------------------------------------------------------
Dviv        aatggctgaataaattgtggtatatgaatactatggaatattactgttctgtaagaaatg
Afla        ------------------------------------------------------------


Shar1       ------------------------------------------------------------
Shar2       ------------------------------------------------------------
Dviv        accaacaggatgatttcagaaaggcctggagagacttacatgaactgatgctgagtgaaa
Afla        ------------------------------------------------------------


Shar1       ------------------------------------------------------------
Shar2       ------------------------------------------------------------
Dviv        tgagcaggaccaggagaacattatatacttcaacaacaatactatatgatgcccagttct
Afla        ------------------------------------------------------------
```

```
Shar1      ------------------------------------------------------------
Shar2      ------------------------------------------------------------
Dviv       gatggacctggccatcctcagcaacgagatcaaccaaatcatttccaatggagcagtaat
Afla       ------------------------------------------------------------


Shar1      ------------------------------------------------------------
Shar2      ------------------------------------------------------------
Dviv       gaactgaaccagctatgcctagagaaagaactttgggagatgacgaaaaaccaatacatt
Afla       ------------------------------------------------------------


Shar1      ------------------------------------------------------------
Shar2      ------------------------------------------------------------
Dviv       gaattcccaatccctatatttatgcccacctgcatatttgatttcctccacaagctaatt
Afla       ------------------------------------------------------------


Shar1      ------------------------------------------------------------
Shar2      ------------------------------------------------------------
Dviv       gcacaatatttcagaatcagattctttttatacagcaaaatatgttttggtcatgaatac
Afla       ------------------------------------------------------------


Shar1      ------------------------------------------------------------
Shar2      ------------------------------------------------------------
Dviv       ttattgtatatctaatttatattttaatgtatttaacatctactggtcatcctgccatct
Afla       ------------------------------------------------------------


Shar1      ------------------------------------------------------------
Shar2      ------------------------------------------------------------
Dviv       aggggaaggggtggggggtgggaggcgaaaaattggaacaagaaagttggcaattgttaa
Afla       ------------------------------------------------------------


Shar1      ------------------------------------------------------------
Shar2      ------------------------------------------------------------
Dviv       tgctgtaaagttatccatgcatataacctgtaaataaaaggctattattaaaaaaaaatt
Afla       ------------------------------------------------------------
```

```
Shar1       -----------------------aaacatttattgtgtctattaaagtaagcaattcatc
Shar2       -----------------------aaacatttattgtgtctattaaagtaagcaattcatc
Dviv        tttttttaaattaaaaaaaaaaaaaacatttattgtgtctattaaagcaagcaattcatc
Afla        -----------------------aaacatttattgtgtctattagagcaagcaattcatc
                                   *********************.**.************

Shar1       aatgcttaatgcccagtgactaactgcccctcctcacaccttcca---------------
Shar2       aatgcttaatgcccagtgactaactgcccctcctcacaccttcca---------------
Dviv        aatgcttaatgccccgtgactaactgcccctcctcacaccttccacagtggtttatttat
Afla        aatgcttaatgccc-atgactgactgcccctcctcacaccttccacaggggtttatttat
            ************** .*****.***********************

Shar1       ------------------------------------------------------------
Shar2       ------------------------------------------------------------
Dviv        tttggttctttttaatgcacatattgtgtgatagttgtgggtaccacatctcccttctag
Afla        tttcgttcttcttaatgctcatattgtgtgatagttgtgggtaccacatctaccttctag


Shar1       ------------------------------------------------------------
Shar2       ------------------------------------------------------------
Dviv        aacatgtgctccctacagatgggattcaggaggtatttaataaatgttcattgtatttgt
Afla        aatatgtgctccctacagatgggattcaggaggtatttaataaatgttcattgtatttgt


Shar1       ------------------------------------------------------------
Shar2       ------------------------------------------------------------
Dviv        tgttctgacatagccagaccgtaacagtaaatgctgctaagcagaggccatccttctgtt
Afla        tgctttgacattgccaggccatagcagtaaatgctgctaagcagaggccatccttctgtt


Shar1       ------------------------------------------------------------
Shar2       ------------------------------------------------------------
Dviv        ttcctccagtcctcccagtgtcttctcatctccccacagttttgtctttttcctttcct
Afla        ttcctccagtcctcccaacatcttctcttctccccacaattttgtctttttcctttcct


Shar1       ------------------------------------------------------------
Shar2       ------------------------------------------------------------
Dviv        cccactgcacttgtctcccctcccctgctttagggctagaccactctgtggctcatgggg
Afla        cccactgcacttgtctcccctctcctgctttagggctagaccactctgtggctcatgggg


Shar1       ------------------------------------------------------------
Shar2       ------------------------------------------------------------
Dviv        tcaccatccagggcagatctcccccactccccttctccccagccttcacacatcccaat
Afla        tcaccatccaaggcagatctcccccacttccccttctccccagccctcacacatcccaat
```

```
Shar1       ------------------------------------------------------------
Shar2       ------------------------------------------------------------
Dviv        cccatcttgacttctggttttctgttctgcttagaagttccaggatgacagctaagcatc
Afla        cccatcttgacttctggttttctcttctgcttagaagttccaggatgacagctaagcatc


Shar1       ------------------------------------------------------------
Shar2       ------------------------------------------------------------
Dviv        tgttccttcccttccttctggcctgcctgtggttcctggctgcctactgccacctggctg
Afla        tgttccttcccttccttctggcctgcctgtggttcctggctgcctactgccacctggctg


Shar1       ------------------------------------------------------------
Shar2       ------------------------------------------------------------
Dviv        aggaagagaaatggagtaaggataggggtctgggaagaagctccatgaacctgcctgact
Afla        aggaagagaaatggagtaaggataggagtctgggaagaagctccatgaacctgcctgact


Shar1       ------------------------------------------------------------
Shar2       ------------------------------------------------------------
Dviv        ttccttctgtgtccatcgtgggtgagtagcctggccagccactatctctgacacaggact
Afla        ttccttctgtgtccatcgtgggtgagtagcctgaccagccaccatctctaacacaggact


Shar1       ------------------------------------------------------------
Shar2       ------------------------------------------------------------
Dviv        agtgtgcagggcgggcctatgatctctatcct----------------gggcttgggctt
Afla        agagtgcaggggggcctatgatcttcctcctgctcccttagcctagagggcttgggctt


Shar1       ------------------------------------------------------------
Shar2       ------------------------------------------------------------
Dviv        cgttttcccctttgggagattccgggtggcctccctcaaatgcagctacctctctcaag
Afla        catttccccttctgggagattctcggtggcctccctcaaatgcagctacctctctcaag


Shar1       ------------------------------------------------------------
Shar2       ------------------------------------------------------------
Dviv        ttctagctattcttgatagtggtgctcactccccatttagtctatagattgggaaccaca
Afla        ttctagctgttcttgatagtggcactcacttcccatttagtctgtagattaggaactaca


Shar1       ------------------------------------------------------------
Shar2       ------------------------------------------------------------
Dviv        agaactgggttccaatacctgagaaaatcacttctgaaacccactctcagcatttcagat
```

```
Afla       agaactgggttccaatacctgagaaaatcacttctgaaacccactctcagcatttcaggt


Shar1      ------------------------------------------------------------
Shar2      ------------------------------------------------------------
Dviv       ttcttcttattaaaaatggggagggggatttaaatgctctctaatgtcctttccagatc
Afla       tccttctttattaaaatggggagggggatttaaatgctctctaaagtcccttccagatc


Shar1      ------------------------------------------------------------
Shar2      ------------------------------------------------------------
Dviv       taaaaattttatgatcccttctactaaatggctacctcccctgccttttcttcagctgct
Afla       taaaaatgttatggttccttctactaaatggctacctcccctgccttttcttcaaccgct


Shar1      ------------------------------------------------------------
Shar2      ------------------------------------------------------------
Dviv       tgccagcctgaagtccctatggagtcacagagccttattaatcaaatctggagaaaagtc
Afla       tatcagcctgaagtccctatggagtcac--agccttattaatcaaatctggagaaaagtc


Shar1      ------------------------------------------------------------
Shar2      ------------------------------------------------------------
Dviv       agatgaatgtaactcctttctatagctaatgttcagcatatgagaggtgagctttaatga
Afla       agatgaatgtaactcctttcaatagccaatgttcagtatatgagaggtgggctttaatga


Shar1      ------------------------------------------------------------
Shar2      ------------------------------------------------------------
Dviv       gacctttgattctacctgaaaaacagggtctagaccaatgagatttcagtgcatctaata
Afla       gacttttgattctacctgaaaaacagggtctagatcaatgagatttcagtgcatctaata


Shar1      ------------------------------------------------------------
Shar2      ------------------------------------------------------------
Dviv       tagattaaatgtgaaattcttctaaacatatttccacatttatcattctgcataagaaaa
Afla       tagattaaatgtgaaattcttctaaacgtatttccacatttatcattttacacaagaaaa


Shar1      ------------------------------------------------------------
Shar2      ------------------------------------------------------------
Dviv       atcagatcaaaaaggggggg--gaaatgagaaagaaaaaagcaagcaagcaacaaaaatga
Afla       atcagatcaaaaagggggggagaaataagaaagaaaaaagcaagcaagcaacaaagatga


Shar1      ------------------------------------------------------------
```

```
Shar2       ------------------------------------------------------------
Dviv        gggagaagcgagatggaaagcaattttattttgatacaattgacttcctttgcaaatctt
Afla        gggagaagcaaggtggaaagcaattttatttagatacaattggcttcctttgcaaatc-t


Shar1       ------------------------------------------------------------
Shar2       ------------------------------------------------------------
Dviv        ttttttttcatgagttgaaaaacattgtgctggaaagggtttacaggcttcggcccagac
Afla        ttttttttcatgagttgaaaaacattatgctggaaagggtttataggtttccccccagag


Shar1       ------------------------------------------------------------
Shar2       ------------------------------------------------------------
Dviv        tccaaaagggccccatggcaaaaaaaaagttaagagcctatctagatagtgctggagact
Afla        tcccaaagggccccatggc--aaaaaaagttaagagcctatctagatagtgctggagact


Shar1       ------------------------------------------------------------
Shar2       ------------------------------------------------------------
Dviv        gagggattaggaaaggtagtctctaagactagtttaaaggtttccacaaacctcttggtc
Afla        aagggattaggaaaggtggtctccaagactagttttgaggtttccacaaacctcttggtc


Shar1       ------------------------------------------------------------
Shar2       ------------------------------------------------------------
Dviv        tcaactgttcccagaacccaaggaaaaaaaaggggcaatt---gaaactccttttgaccc
Afla        tcaactgttccaagaacccaaggaaaaaaaaggggcaattaaagaaactccctttg----


Shar1       ------------------------------------------------------------
Shar2       ------------------------------------------------------------
Dviv        tgagatactaggaggggaatgccaaaagtggacaagaatcaaatttcttttctgtctagc
Afla        ------------------------------------------------------------


Shar1       ------------------------------------------------------------
Shar2       ------------------------------------------------------------
Dviv        ctgtaagacttcctctattctcaggctttctcctctttgtttgtgaggcacttacctcaa
Afla        ------------------------------------------------------------


Shar1       ------------------------------------------------------------
Shar2       ------------------------------------------------------------
Dviv        tccctggcctttgctcttttggctcctctagcattgaaatggtcaggcaaaaatggtcag
Afla        ------------------------------------------------aaaaaaagtc--
```

```
Shar1       ----------------------------------------------------------ca
Shar2       ----------------------------------------------------------ca
Dviv        gggtctccactcctgcctccgggagacccccttcccagaggtctccctcttcccaattca
Afla        ---tctccacccctccctccgggagactcccttcccagaggcctccctcttcccaattca
                                                                      **

Shar1       gtctatccctggccctgccagtcagacatatcttctcttcattttgttttctttaaagca
Shar2       gtctatccctggccctgccagtcagacatatcttctcttcattttgttttctttaaagca
Dviv        gtctatccctggccctgccagtcagacatatcttcttttcattttgttttctttgaagca
Afla        gcctatccctggccctgccagtcagacatatcttctcttcattttgttttctttgaagca
            *.**********************************.*****************.*****

Shar1       ctgaacaaaaaatccaagaagaacatcaggaaagagatagaaaccaagaaatcttccgag
Shar2       ctgaacaaaaaatccaagaagaacatcaggaaagagatagaaaccaagaaatcttccgag
Dviv        ctgaacaaaaaatccaagaagaacatcaggaaagagatagaaaccaagaaatcctccgag
Afla        ctgaacaaaaaatccaagaagagcatcaggaaagagatagaaaccaagaaatcctccgag
            *********************.*******************************.******
```
