# Supplementary material for: Genome of the endangered eastern quoll (Dasyurus viverrinus) reveals signatures of historical decline and pelage color evolution
Source: Commun Biol. 2024 May 25;7:636. doi: 10.1038/s42003-024-06251-0 (PMC11128018; doi:10.1038/s42003-024-06251-0)
Supplement: Supplementary file 6 — Reporting Summary [file 42003_2024_6251_MOESM6_ESM.pdf]

nature portfolio

Corresponding author(s): Charles Feigin

Last updated by author(s): Apr 3, 2024

Reporting Summary

Nature Portfolio wishes to improve the reproducibility of the work that we publish. This form provides structure for consistency and transparency in reporting. For further information on Nature Portfolio policies, see our Editorial Policies and the Editorial Policy Checklist.

Statistics

For all statistical analyses, confirm that the following items are present in the figure legend, table legend, main text, or Methods section.

n/a	Confirmed	
☐	☒	The exact sample size (*n*) for each experimental group/condition, given as a discrete number and unit of measurement
☐	☒	A statement on whether measurements were taken from distinct samples or whether the same sample was measured repeatedly
☒	☐	The statistical test(s) used AND whether they are one- or two-sided<br>*Only common tests should be described solely by name; describe more complex techniques in the Methods section.*
☒	☐	A description of all covariates tested
☒	☐	A description of any assumptions or corrections, such as tests of normality and adjustment for multiple comparisons
☒	☐	A full description of the statistical parameters including central tendency (e.g. means) or other basic estimates (e.g. regression coefficient) AND variation (e.g. standard deviation) or associated estimates of uncertainty (e.g. confidence intervals)
☒	☐	For null hypothesis testing, the test statistic (e.g. *F*, *t*, *r*) with confidence intervals, effect sizes, degrees of freedom and *P* value noted<br>*Give P values as exact values whenever suitable.*
☒	☐	For Bayesian analysis, information on the choice of priors and Markov chain Monte Carlo settings
☒	☐	For hierarchical and complex designs, identification of the appropriate level for tests and full reporting of outcomes
☒	☐	Estimates of effect sizes (e.g. Cohen's *d*, Pearson's *r*), indicating how they were calculated

Our web collection on statistics for biologists contains articles on many of the points above.

Software and code

Policy information about availability of computer code

Data collection	N/A
Data analysis	Software: Hifiasm v0.15.4-r34, purge_dups v1.2.5, HiRise-e0moc, BWA-0.7.17, Pairtools v1.0.2, juicer_tools v 1.22.01, Juicebox v1.11, bbmap v39.01, BUSCO v5.4.6, Liftoff v1.6.3, Trimmomatic v0.39, funannotate v1.8.14, eggNOG-mapper web server, RepeatMasker v4.1.3, Bedtools v2.29.0, HapCUT2 v1.2, MAFFT web server, minimap2 v2.24, samtools v1.11, DeepVariant v1, MSMC2 v2.1.4, MACSE v2, IGV v2.12.3, observations_map.r, ideogram.r.

For manuscripts utilizing custom algorithms or software that are central to the research but not yet described in published literature, software must be made available to editors and reviewers. We strongly encourage code deposition in a community repository (e.g. GitHub). See the Nature Portfolio guidelines for submitting code & software for further information.

Data

Policy information about availability of data

All manuscripts must include a data availability statement. This statement should provide the following information, where applicable:

- Accession codes, unique identifiers, or web links for publicly available datasets
- A description of any restrictions on data availability
- For clinical datasets or third party data, please ensure that the statement adheres to our policy

The eastern quoll reference genome and all sequence data used in its generation are available on NCBI under BioProject PRJNA758704. Transcriptome data used to

produce gene annotations are available under BioProject PRJNA963007. Gene annotation GFF files and all original code used in this study can be accessed in a permanent FigShare repository: https://figshare.com/s/29cdab4b5d5cde2c4d55.

Research involving human participants, their data, or biological material

Policy information about studies with human participants or human data. See also policy information about sex, gender (identity/presentation), and sexual orientation and race, ethnicity and racism.

Reporting on sex and gender	N/A
Reporting on race, ethnicity, or other socially relevant groupings	N/A
Population characteristics	N/A
Recruitment	N/A
Ethics oversight	N/A

Note that full information on the approval of the study protocol must also be provided in the manuscript.

Field-specific reporting

Please select the one below that is the best fit for your research. If you are not sure, read the appropriate sections before making your selection.

☒ Life sciences ☐ Behavioural & social sciences ☐ Ecological, evolutionary & environmental sciences

For a reference copy of the document with all sections, see nature.com/documents/nr-reporting-summary-flat.pdf

Life sciences study design

All studies must disclose on these points even when the disclosure is negative.

Sample size	Reference genome was produced for a single individual from a captive breeding colony.
Data exclusions	No data were excluded.
Replication	A single individual had DNA sequenced to produce the genome assembly. One replicate of each of five tissues had RNA-Seq conducted to annotate the genome. ASIP deletion was verified in two Tasmanian devil genome assemblies from separate individuals.
Randomization	N/A
Blinding	N/A

Reporting for specific materials, systems and methods

We require information from authors about some types of materials, experimental systems and methods used in many studies. Here, indicate whether each material, system or method listed is relevant to your study. If you are not sure if a list item applies to your research, read the appropriate section before selecting a response.

Materials & experimental systems

n/a	Involved in the study	
☒	☐	Antibodies
☐	☒	Eukaryotic cell lines
☒	☐	Palaeontology and archaeology
☐	☒	Animals and other organisms
☒	☐	Clinical data
☒	☐	Dual use research of concern
☒	☐	Plants

Methods

n/a	Involved in the study	
☒	☐	ChIP-seq
☒	☐	Flow cytometry
☒	☐	MRI-based neuroimaging

Eukaryotic cell lines

Policy information about cell lines and Sex and Gender in Research

Cell line source(s)	Primary fibroblasts were isolated from the skin of a female eastern quoll (Dasyurus viverrinus) that was euthanised for veterinary reasons in a captive breeding colony as secondary use.
Authentication	RNA extracted from cells was aligned to the eastern quoll genome.
Mycoplasma contamination	Cells were not tested for mycoplasma
Commonly misidentified lines (See ICLAC register)	N/A

Animals and other research organisms

Policy information about studies involving animals; ARRIVE guidelines recommended for reporting animal research, and Sex and Gender in Research

Laboratory animals	The study did not involve laboratory animals
Wild animals	No wild animals were use for this study. Materials were collected from a female eastern quoll decended from wild animals, belonging to the captive breeding program at Aussie Ark sanctuary that was euthanized for veterinary reasons as secondary use.
Reporting on sex	The female was verified as female by veterinarians at Aussie Ark and Taronga Zoo as part of routine procedures in the captive breeding program.
Field-collected samples	N/A
Ethics oversight	Samples were collected under the auspices of the Taronga Conservation Society Australia's Animal Ethics Committee (approval no. 3b1218), pursuant to NSW Office of Environment and Heritage-issued 543 scientific license no. SL100104..

Note that full information on the approval of the study protocol must also be provided in the manuscript.

Plants

Seed stocks	N/A
Novel plant genotypes	N/A
Authentication	N/A
